# Amplitude of low-frequency fluctuations in first-episode, drug-naïve depressive patients: A 5-year retrospective study

**DOI:** 10.1371/journal.pone.0174564

**Published:** 2017-04-06

**Authors:** Kerang Zhang, Zhifen Liu, Xiaohua Cao, Chunxia Yang, Yong Xu, Ting Xu, Cheng Xu, Zhi Yang

**Affiliations:** 1 Department of Psychiatry, First Hospital of Shanxi Medical University, Taiyuan, China; 2 Key Laboratory of Behavioral Science and Magnetic Resonance Imaging Research Center, Institute of Psychology, Chinese Academy of Sciences, Beijing, China; 3 Laboratory for Functional Connectome and Development, Institute of Psychology, Chinese Academy of Sciences, Beijing, China; 4 Department of Radiology, Shanxi Provincial Hospital, Taiyuan, China; University of Oxford, UNITED KINGDOM

## Abstract

Despite different treatments and courses of illness, depressive symptoms appear similar in bipolar disorder (BD) and major depressive disorder (MDD), causing BD with an onset of depressive episode being frequently misdiagnosed as MDD, and leading to inappropriate treatment and poor clinical outcomes. Therefore, there is an urgent need to explore underlying neural basis to distinguish BD from MDD. The medical records of 80 first-episode, drug-naïve depressive patients with an initial diagnosis of MDD and illness duration of at least 5 years were reviewed retrospectively for this study. Fourteen bipolar depressed patients with a diagnosis conversion from MDD to BD, 14 patients with diagnosis of MDD, and 14 healthy subjects demographically matched with the BD group, were selected to participate in the study. Firstly, we examined whether there were differences among the three groups in whole brain fALFF during resting state. Secondly, clusters showing group differences in fALFF in any two groups were chosen as regions of interest (ROI) and then correlation between clinical features and fALFF values of ROIs were calculated. The BD group showed increased fALFF in bilateral putamen relative to both the MDD group and controls, while the MDD group exhibited decreased fALFF in left superior frontal gyrus (SFG) relative to both the BD group and controls (*p* < 0.05, corrected). Positive correlations between abnormality in the putamen and symptom severity were observed (significant for the MDD group, *p =* 0.043; marginally significant for the BD group, *p* = 0.060/0.076). These results implicate that abnormalities of key regions in the striatum and prefrontal areas may be trait markers for BD and MDD.

## Introduction

Differences in clinical features between major depressive disorder (MDD) and bipolar disorder (BD) have been confirmed by epidemiologic and clinical studies. For example, compared with MDD, BD were characterized by lower lifelong prevalence (2.1% vs. 16.2%) [1,2], younger median age of onset (25 vs. 32 years) [3], and more depressive episodes [4]. However, it is very difficult to distinguish MDD from BD, partially due to the fact that both of them show similar depressive episodes. It has been showed that approximately 60% of bipolar patients seeking treatment for depression are misdiagnosed as MDD or recurrent unipolar depression (UD) [5,6], leading to inappropriate prescription of antidepressants, increasing rate of switching to mania, and greater health care costs [7–9]. Therefore, there is a critical need to develop clinically objective biomarkers that could be used to discriminate BD from MDD.

Functional magnetic resonance imaging (fMRI) has been applied widely to identify potential neuropathphysiology of BD or MDD. Chen et al. [10] systematically reviewed the task-based fMRI literature on BD and found that BD exhibited underactivity in the inferior frontal cortex (IFG) and putamen, and overactivity in limbic areas (parahippocampal gyrus, hippocampus, amygdala and basal ganglia) relative to healthy controls. A meta-analysis [11] found that MDD showed overreactive salience network (amygdala, dorsal anterior cingulate cortex, and insula) and low response of dorsolateral prefrontal cortex and dorsal striatum to negative information. In addition, different brain activation patterns have been observed by directly comparing activations BD and MDD during performing a series of tasks (e.g., executive function [12,13], facial expressions [14–16], and reward [17]). However, the findings of these studies vary from the tasks performed by participants, posing a challenge to compare data obtained from different cognitive studies.

In the past decade, attention has been focused on functional abnormalities during task-free resting state in the neuroimaging field. The pioneering study by Biswal et al. [18] has revealed that spontaneous low frequency (0.01 ~ 0.08 Hz) fluctuations (LFF) in the resting state blood oxygen level dependent (BOLD) signal were highly synchronous among left and right motor cortices (i.e., resting state functional connectivity, RSFC). Abnormal LFF synchrony among remote brain areas has been reported in MDD (e.g., abnormal RSFC in cortico-limbic mood regulating circuit and default mode network [19–22]) and BD (e.g., disconnection in fronto-limbic circuit [23]). Moreover, different RSFC patterns have been found between BD and MDD in resting-state fMRI studies (e.g., [24–27]). However, these studies explored LFF from the perspective of temporal synchronization (i.e., RSFC), but neglected regional activity of MDD and BD during resting state.

The amplitude of LFF (ALFF) of BOLD signal, an index developed by [28] and calculated from the square root of power spectrum, was integrated in a low-frequency range, and reflects regional spontaneous neural activity of the brain. ALFF has been used widely to measure aberrant regional brain responses of various psychiatric disorders (e.g., ADHD [28], schizophrenia [29], BD [30], MDD [31,32]) during resting state. Since ALFF is sensitive to the physiological noise, Zou et al. [33] proposed an improved index named fractional ALFF (fALFF) approach, i.e., the ratio of power spectrum of low frequency to that of the entire frequency range. Herein, given the results of previous neuroimaging studies investigating similarities and discrepancy between BD and UD, we hypothesized that differences in fALFF may be found in regions belonging to cortical-limbic circuit and basal ganglia between first-episode medication-naïve depressed patients who later experienced manic/hypomanic episodes and those who never experienced manic/hypomanic episodes for same period of time. This study investigated the differences in fALFF between bipolar depressed patients with a diagnosis conversion from MDD to BD at the end of follow-up care period, major depressive patients with persistent diagnosis of MDD at the end of follow-up care period, and healthy controls in fALFF using a data-driven image analysis method. Apart from comparing fALFF between different clinical groups, we would further investigate the associations between clinical features and fALFF value of the regions showing group differences, and attempt to evaluate the potential of these regions to distinguish different groups.

## Materials and methods

Written informed consent was obtained from each participant and consent from each participant’s guardian was also obtained prior to data acquisition. This study was approved by the Ethical Committee for Medicine of First Hospital of Shanxi Medical University, China.

### Participants

First, we conducted a retrospective study that reviewed the medical records of 80 first-episode, drug-naïve depressive patients who were hospitalized in Department of Psychiatry, First Hospital of Shanxi Medical University in Taiyuan, China, between July 1, 2007 and December 31, 2009, with an initial diagnosis of MDD and no history of mania or hypomania. MDD diagnosis was based on the Chinese version of the Modified Structured Clinical Interview for DSM-IV, patient version (SCID-I/P) [34].

All participants (patients and health controls) included in this study needed to meet the following criteria: 1) be aged between 18 and 50 years; 2) right-handedness; 3) having no history of neurological illnesses or other severe diseases; and 4) having no history of head injury. Beyond that, the patient participants needed to satisfy four additional including criteria: 1) in their first episode and drug-naïve when they received the MRI scanning; 2) having at least 5 years of follow-up care as of January 1, 2015; 3) having been diagnosed with MDD or BP at the end of the 6 years follow-up; and 4) having a total score of 17-item Hamilton Depression rating scale (HAMD) not less than 17 during scanning. Exclusion criteria for the patients included comorbid neurological or psychological disorders, a diagnosis of a mood disorder due to a general medical condition, or a diagnosis that converted from MDD to another diagnosis other than BP. Half of the 80 patients met the aforementioned criteria.

Then, we followed up these 40 patients prospectively. They were reviewed every six month, the SCID-I/P diagnosis was redone during every visiting. After > 5 year follow-up care (range 5.02~7.40 years, 6.23 ± 0.66 years), 14 of them had experienced at least one manic/hypomanic episode, and their diagnosis was converted from MDD to BD (BD group, n = 14), 36 of them had experienced at least two major depressive episodes and had never experienced manic/hypomanic episodes. We further chose a subgroup of 14 patients from 36 MDD patients, matched age, gender and education-level with the BD group (MDD group, n = 14). There were no history of psychiatric illness in the health control participants and their first-degree relatives. The controls were demographically matched with the BD group (CON group, n = 14). Written informed consent was obtained from each participant and consent from each participant’s guardian was also obtained prior to data acquisition. The Ethical Committee for Medicine of the First Hospital of Shanxi Medical University approved this study.

### MRI acquisition

Images were acquired using a Siemens Trio 3-Tesla scanner (Siemens, Erlangen, Germany). Foam pads and earplugs were used to limit head motion and reduce scanner noise. For each subject, resting-state functional data and a high-resolution T1-weighted anatomical image were acquired. Scanning parameters of the functional and structural images are as follows: 1) echo-planar imaging (EPI) sequence with 32 axial slices, repetition time (TR) = 2000 ms, echo time (TE) = 30 ms, thickness/skip = 3/1 mm, field of view (FOV) = 240×240 mm^2^, matrix = 64×64 mm^2^, flip angle (FA) = 90°, 212 volumes; 2) 3D T1-weighted magnetization-prepared rapid gradient echo (MPRAGE) sequence with 160 sagittal slices, TR = 2300 ms, TE = 2.95 ms, thickness/skip = 1.2/0.6 mm, FOV = 225×240 mm, matrix = 240×256 mm, FA = 9°, 160 volumes. During the resting state scan, subjects were instructed to close their eyes, and remain awake and as motionless as possible. After the resting state scans, all subjects were confirmed that they did not fall asleep during the scan.

### Preprocessing and calculation of fALFF

Preprocessing and statistical analysis of the imaging data were carried out using Connectome Computation System (CCS: http://lfcd.psych.ac.cn/ccs.html) [35], which combines multiple modules MRI to provide a computational platform for brain connectome analysis. The data preprocessing was composed of anatomical and functional processing steps. The structural preprocessing steps included: 1) removal of MR image noise using a spatially adaptive non-local means filter [36,37]; 2) brain extraction using a hybrid watershed/surface deformation procedure; 3) automated segmentation of cerebrospinal fluid (CSF), white matter (WM) and deep gray matter (GM) volumetric structures as well as surface reconstruction; and 4) spatial normalization of individual anatomical images to MNI152 standard brain template. The functional preprocessing steps included: 1) removing the first 5 volumes from each scan to allow for signal equilibration; 2) despiking and slice timing correction; 3) motion correction; 4) 4D grand mean intensity normalization; 5) aligning individual functional images to individual anatomical image using the grey-white matter boundary-based registration (BBR) algorithm [38]; 6) registering individual functional images to MNI152 standard template; 7) regressing out the Friston-24 motion time series and mean signals of WM and CSF to reduce the effects of these confounding factors [39, 40]; 8) removing linear and quadratic trends; and 9) spatial smoothing with Gaussian Kernel (FWHM = 6 mm).

Following the preprocessing steps, a quality control procedure (QCP) [41] was conducted to ensure the quality of images. In brief, QCP produces screenshots for visually inspecting the quality of brain extraction, segmentation, cortical surface reconstruction, BBR-based registration and head motion. Additionally, the minimal cost value of boundary based registration (mcBBR) and the mean frame-wise displacement (mean FD) [42] were calculated for measuring registration quality and head motion, which were used as covariates in the following group analysis. One patient in the MDD group was excluded due to corruption of the resting-state fMRI data. Finally, 14 in the BD group, 13 in the MDD group and 14 in the CON group passed the QCP. More details about QCP can be found at http://lfcd.psych.ac.cn/ccs.html.

Subsequently, fALFF was computed in individual volume space within CCS. The amplitudes were calculated as square root of the power spectrum obtained at each frequency point. fALFF were calculated as the sum of amplitude in the range of 0.01 ~ 0.1 Hz divided by the total amplitude in the entire detectable frequency range, i.e., 0 ~ 0.25 Hz. The details of computation can be found in Zou et al. [33].

### Statistical analysis

#### Demographic and clinical data comparisons

One-way analysis of variance (ANOVA) was performed to examine whether there were differences among the three groups in age and years of education. Pearson Chi-square test was used to compare gender ratios. According to the distribution of data, two-sample t-test or Mann-Whitney U test were conducted to compare the total score of HAMD and the duration of illness between the two patient groups.

#### Whole brain fALFF analysis

Group-level analyses of fALFF were conducted with FLAME in FSL using ordinary least squares (OLS) model. ANCOVA was first performed to investigate the differences among three groups followed by two-sample t-test for any two groups (BD-CON, MDD-CON and BD-MDD). Age, gender were included as covariates in all the group comparison models. In addition, mean FD and the minimal cost value during BBR (mcBBR) were applied as covariates to control the influences of registration and head motion. The resulting fALFF maps of group differences were threshold by Z > 2.3, corrected *p* < 0.05 using Gaussian Random Field theory. The significant clusters were defined as region of interested (ROIs) and extracted for the following analyses.

#### ROI-wise fALFF analysis

The clusters showing significant differences in any two groups were defined as regions of interest (ROI). The mean fALFF values of the ROIs were extracted for each participant. Then one-way ANOVA and post-hoc analyses were performed respectively to compare differences of mean fALFF among the three groups and between each of the two groups.

#### Correlations with clinical variables

In order to investigate the relationship between clinical variables and regional spontaneous brain activity during resting state, correlation analyses were performed between total score of HAMD, illness duration and the mean fALFF value of each ROI in two patient groups, controlling for the effect of age, gender and years of education. A two-tailed *p* level of 0.05 was used as the criterion of statistical significance.

## Results

### Demographic and clinical data comparisons

Three groups were comparable in age, gender and years of education. There were no significant differences between the BD and MDD group in total score of HAMD and illness duration. Details of the results were listed in Table 1.

**Table 1:** Demographic and clinical characters for the BD, MDD and the Control group.

Variable	BD (n = 14)	MDD (n = 13)	CON (n = 14)	Statistical value	p-value
	Mean (SD)	Mean (SD)	Mean (SD)		
**Age**	33.79 (11.08)	33.46 (9.49)	34.21 (10.74)	0.018 ^a^	0.983
**Gender (Male/Female)**	6/8	6/7	6/8	0.039 ^b^	0.981
**Education (years)**	11.71 (3.05)	11.77 (3.70)	13.14 (3.88)	0.717 ^a^	0.495
**Total score of HAMD**	18.54 (5.21)	20.15 (3.24)	—	-0.95 ^c^	0.352
**Illness duration (months)**	14.14 (14.52)	9.14 (5.13)	—	-0.253 ^d^	0.800

BD: bipolar disorder; MDD: major depression disorder; CON: control; SD: standard deviation; HAMD: Hamilton Depression rating scale.

a: F-value for one-way ANOVA

b: χ2-value for chi-square test

c: t-value for two-sample t-test

d: Z-value for Mann-Whitney U test.

### Whole brain fALFF analysis

**Fig 1:**
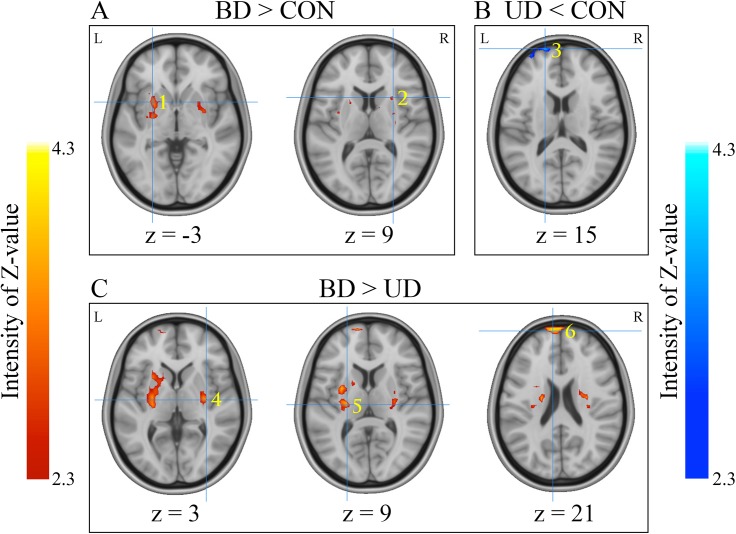
Regions showing significant differences in fALFF between each of the two groups. **(A)** demonstrates regions showing group difference between the BD and CON groups, with the fALFF value increased in BD relative to the controls. **(B**) demonstrates the region exhibiting group difference between the MDD and CON group, with the fALFF value increased in the CON group relative to MDD.**(C**) demonstrates regions showing group difference between the BD and MDD groups, with the fALFF value increased in BD relative to MDD. The color bar means the intensity of Z-value, yellow/red in the color bar denotes relatively higher fALFF values, and cyan /blue in the color bar denotes relatively higher fALFF values. Locations of the clusters showing group differences were indicated by Arabic numbers labeled in each image. The number in the lower right corner of each image refers to the z MNI coordinates. Brain region labels: 1. left putamen; 2. right putamen; 3. left superior frontal gyrus/medial frontal gyrus; 4. right putamen; 5. left putamen; 6. left superior frontal gyrus. Left in the figure indicates the right side of the brain. BD: bipolar disorder. MDD: major depression. CON: control.

One-way ANOVA revealed significant differences in left and right putamen as well as left superior frontal gyrus (SFG) among three groups (*p* < 0.05, corrected) (Table 2). Then two-sample t-tests were performed for each pair of groups. For the BD vs. CON comparison, significantly increased fALFF in bilateral putamen was observed in the BD group relative to the CON group. For the MDD vs. CON comparison, MDD showed significantly lower fALFF in left SFG extending to medial frontal gyrus (MFG) than CON group. For the BD vs. MDD comparison, the BD group exhibited significantly increased fALFF values in left SFG and bilateral putamen relative to the MDD group. Details of the results are given in Fig 1 and Table 2.

**Table 2:** Regions showing significant differences in fALFF among the groups.

Area	Cluster Size (voxels)	BA	Side	MNI (peak)			Z-value (peak)
				x	y	z	
**One-way ANOVA among three groups**							
Putamen	38		R	33	-12	3	3.10
Putamen	122		L	-24	-18	9	3.32
SFG/MFG	60	10	L	-18	69	15	4.04
SFG	60	8	L	-30	42	45	3.58
**Two-sample t-tests between each two groups**							
**BD > CON**							
Putamen	180		L	-27	9	-3	3.42
Putamen	100		R	27	15	9	3.15
**MDD < CON**							
SFG/MFG	121	10	L	-18	69	15	4.26
**BD >MDD**							
Putamen	171		R	33	-12	3	3.52
Putamen	340		L	-24	-18	9	3.86
SFG	163	10	L	-9	66	21	4.04

A minimum Z > 2.3 and a corrected *p* < 0.05 at the cluster level were set at the threshold.

fALFF: fractional amplitude of low-frequency fluctuation; BA: Brodmann area; MNI: Montreal Neurological Institute; BD: bipolar disorder; MDD: major depressive disorder; CON: control; SFG: superior frontal gyrus; MFG: medial frontal gyrus; L: left; R: right.

### ROI-wise fALFF analysis

Clusters showing significant group differences were chosen as ROI to verify fALFF differences across groups. There were six ROIs in total (see Fig 1 and Table 2): 1) left putamen resulted from the BD-CON comparison (L PUTBD-CON); 2) right putamen resulted from the BD-CON comparison (R PUTBD-CON); 3) left SFG/MFG resulted from the MDD-CON comparison (L SFG MDD-CON); 4) right putamen resulted from the BD-MDD comparison (R PUTBD-MDD); 5) left putamen resulted from the BD-MDD comparison (L PUTBD-MDD); and 6) left SFG resulted from the BD-MDD comparison (L SFG BD-MDD).

Subsequently, a mean fALFF value was calculated by averaging fALFF values of all the voxels in each ROI individually. One-way ANOVA revealed significant differences among the three groups in every ROI: L PUT BD-CON, *F* = 9.191, *p* = 0.001; R PUT BD-CON, *F* = 7.897, *p* = 0.001; L SFG MDD-CON, *F* = 13.091, *p* < 0.001; R PUT BD-MDD, *F* = 8.734, *p* = 0.001; L PUT BD-MDD, *F* = 10.088, *p* < 0.001; L SFG BD-MDD, *F* = 15.972, *p* < 0.001.

**Fig 2:**
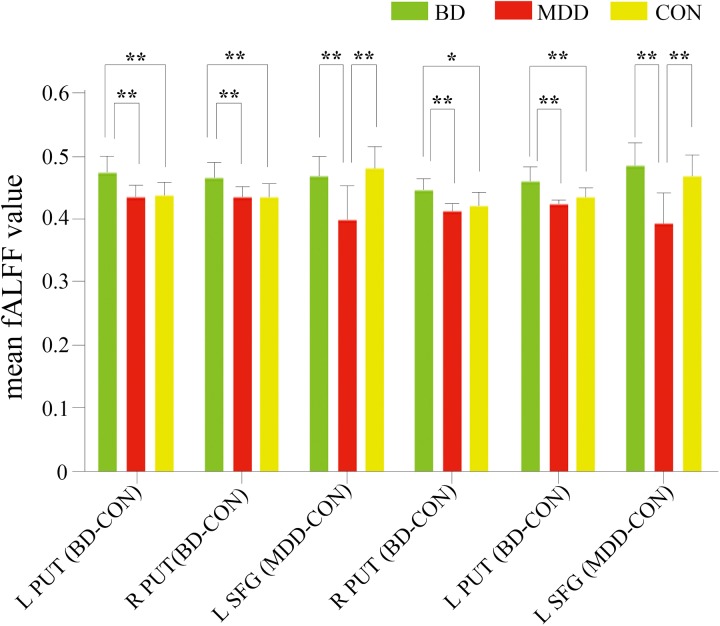
Comparisons of mean fALFF value in each ROI across groups generated from post hoc analysis using Bonferroni correction. Distinct ROIs were presented in the X-axis. The Y-axis represented mean fALFF value of each ROI. A single asterisks means a significance level of p < 0.05, and double asterisks indicate a significance level of *p* <0.01. BD: bipolar disorder. MDD:. CON: control. L PUT BD-CON: left putamen resulted from BD vs. CON comparison; R PUT BD-CON: right putamen resulted from BD vs. CON comparison; L SFG MDD-CON: left SFG/medial frontal gyrus resulted from MDD vs. CON comparison; R PUT BD-MDD: right putamen resulted from BD vs. MDD comparison; L PUT BD-MDD: left putamen resulted from BD vs. MDD comparison; L SFG BD-MDD: left SFG resulted from BD vs. MDD comparison.

Then post-hoc analyses using Bonferroni correction were performed. We found that the BD group showed significantly higher mean fALFF value in bilateral putamen than both the MDD group (L PUT BD-CON, *p* = 0.002; R PUT BD-CON, *p* = 0.002; L PUT BD-MDD, *p* < 0.001; R PUT BD-MDD, *p* = 0.001) and CON group (L PUT BD-CON, *p* = 0.002; R PUT BD-CON, *p* = 0.003; L PUT BD-MDD, *p* = 0.005; R PUT BD-MDD, *p* = 0.019). In addition, the MDD group demonstrated significantly lower mean fALFF value in left superior frontal area than both the BD group (L SFG MDD-CON, *p* = 0.001; L SFG BD-MDD, *p* < 0.001) and CON group (L SFG MDD-CON, *p* < 0.001; L SFG BD-MDD, *p* < 0.001). These results are presented in Fig 2.

### Correlations with clinical variables

Partial correlation analyses were utilized to investigate possible association between clinical characteristics and mean fALFF of the ROI showing significant group differences, controlling for the effect of age, gender and education level. No significant correlation was found between illness duration and mean fALFF of the six ROIs. However, in the MDD group, significant positive correlation was found between the total score of HAMD and mean fALFF of left putamen (L PUT BD-MDD, *r* = 0.647, *p* = 0.043). Besides, in the BD group, marginally significant positive correlations were found between the total score of HAMD and the mean fALFF value of bilateral putamen (L PUT BD-CON, *r* = 0.583, *p* = 0.060; the R PUT BD-CON, *r* = 0.555, *p* = 0.076).

## Discussion

In the present study, we retrospectively studied a group of first-episode medication-naïve patients with a diagnosis of MDD. For at least 5 years after onset of the illness, one subgroup was diagnosed as BD and the other subgroup maintained the diagnosis of MDD. We examined the resting-state fALFF within the whole brain to identify brain regions displaying activity differences among the three groups. We observed that, compared to both the MDD and CON, the BD group showed increased fALFF in bilateral putamen, while the MDD group exhibited decreased fALFF in left SFG relative to the other two groups (*p < 0*.*05*, corrected). Subsequent ROI analyses confirmed that these regions reflected brain abnormalities specific to different groups. ROC analysis further revealed acceptable performance (with sensitivity and specificity higher than 70% in general) of the putamen and SFG in identifying different groups. Finally, correlation analysis showed that significant positive correlations between fALFF in the putamen and symptom severity of MDD group (*r* = 0.647, *p = 0*.*043*).

### Distinct bilateral putamen involvement in BD and MDD

Compared with both the MDD and CON groups, the BD group showed significantly increased fALFF in bilateral putamen, and no such difference was found between MDD and the CON groups. As an important component of the striatum, the putamen belongs to the lateral paralimbic system [43] and is interconnected with medial prefrontal cortex [44]. Through functional connection with prefrontal cortex and subcortical limbic regions, the putamen is considered to be involved in processing emotional and motivational information [45,46].

Functional and structural abnormalities of the putamen in bipolar disorder have been reported in a number of studies. For fMRI studies, increased activation of the putamen has been observed in the BD patients during both task performing (e.g., emotional processing [47,48], response inhibition [49, 50], working memory [20, 51] and resting state (e.g., increased ALFF [30]). For structural MRI studies, enlargement of the putamen has been commonly found in bipolar patients (e.g., [52–56], except for [57,58]). More importantly, there were evidences showed that this putamen abnormality in function (e.g., higher activity during performing emotional task [48]) and structure (e.g., increased volume [53]) among BD patients is not mood state-dependent, but trait-related. Additionally, greater activation of the putamen was found in various age groups (e.g., pediatric [51], adolescents [54]) and relatives (e.g., first-degree relatives [47], offspring [59] of the BD patients. A recent meta-analysis identified distinct patterns of basal ganglia (especially putamen and caudate) engagement for BD and MDD within the facial affect processing network [60]. Specifically, BD patients expressed increased activation in the putamen and in the caudate in response to negative and positive facial expressions, respectively. In addition, patients with basal ganglia lesions may develop both manic and depressive symptoms [61, 62], suggesting possible role of putamen in the pathophysiology of BD.

Based on the above evidences and our findings, we suggested that the abnormality of the putamen might be a trait marker of BD, which might occur early in the course of the BD, and can be observed in bipolar patients in different clinical episode.

### Distinct left SFG involvement in BD and MDD

The MDD group exhibited significantly decreased fALFF in left SFG relative to both the BD group and CON group, and no such difference was found between BD and the CON groups. SFG consists of the dorsal and medial prefrontal regions, and they were core nodes of executive control network (ECN) and default mode network (DMN), respectively [63]. Moreover, previous studies have showed that the superior frontal white matter links the dorsolateral prefrontal cortex (a hub node of ECN) and anterior cingulate cortex (a hub node of DMN) with subcortical nuclei [64]. We speculate that the dysfunction of SFG might hinder information exchange between internal (relative to DMN) and external (relative to ECN) environments, causing MDD to show a series of defects in mood management and cognitive control.

In line with our finding, both functional and structural abnormalities of the SFG among MDD patients have been observed in a large body of researches. Decreased activation in the SFG has been observed in the MDD patients during both task performing (e.g., reward processing [65]) and resting state (e.g., decreased ALFF [31]). Reduced local functional connectivity of SFG was found in first-episode, drug-naïve [66], treatment-resistant, and treatment-sensitive patients with depression [67]. Moreover, our finding was supported by two recent meta-analyses. These meta-analysis were focused on task-based fMRI [68] and resting-state [69] studies, respectively, and the authors observed underactivation and underconnectivity in SFG among patients with MDD. In structural MRI studies, decreased volume of SFG has been reported consistently among MDD patients with different clinical characteristics (e.g., first-episode, medication-naïve MDD [70], treatment-resistant depression [71,72], remitted depression [73]).

Our subsequent ROC analysis showed SFG had better performance in discriminating BD from MDD and CON, with the AUC of the two ROIs (L SFG MDD-CON and L SFG BD-MDD) were 0.907 and 0.929, respectively. Consistent with our finding, two previous studies have observed that SFG could be used to classify MDD and healthy controls [69,74]. Based on the above evidences and our findings, we suggest that decreased activity in the left SFG might be associated with MDD.

### Putamen activity association with depression severity

Correlation analyses revealed possible association between the fALFF value of putamen and total score of HAMD in MDD (left putamen, *r* = 0.647, *p* = 0.043) and BD (marginal significant, left putamen, *r* = 0.583, *p* = 0.060; right putamen, *r* = 0.555, *p* = 0.076) groups, indicating that the higher activity of the putamen in resting state, the more serious depressive symptoms in two patient groups. The basal ganglia (including putamen) might be involved in emotion control, and disrupting in basal ganglia-cortical connectivity may contribute to depressive symptom expression [75,76]. Because of the functional connection with prefrontal cortex and subcortical limbic regions, the putamen is considered to be involved in emotional and motivational processing [45,46].

As stated above, a large number of studies have reported that functional and structural abnormalities of the putamen in various age, different mood states of BD groups, ever relatives of BD patients also showed dysfunction in putamen. In contrast, there were only a few studies reported functional and structural abnormalities in the putamen among MDD patients. A structural MRI study observed that depressed patients had significantly smaller putamen nuclei compared with controls [77]. The finding from a PET research showed that extracellular dopamine in bilateral putamen is lower in MDD subjects exhibiting motor retardation [78].

Although our results suggest that there is a potential clinical significance of putamen in mood disorders, they also raised a couple of questions to be addressed in future studies. For example, what happens in communicating between putamen, as a component of basal ganglia-cortical circuits, with other parts of the circuits among mood disorders? What are the common and distinct putamen engagements in the circuits between BD and MDD? Further structural and functional connectivity researches are warrant in this regard.

### Limitations

Several limitations of the current study should be addressed. Firstly, the sample size of each group was relatively small. Nevertheless, given the difficulty to obtain enough retrospective data, the evidence for early identification of BD provided by our results of the present study is valuable. With the continuation of our longitudinal study, analytical results based on larger sample size and stricter analysis (eg, multiple comparison correction) will be more reliable and more applicable for clinical practice. Secondly, the medical records of 80 first-episode, drug-naïve depressive patients with an initial diagnosis of MDD and illness duration of at least 5 years were retrospected for this study. Although this is quite a long period, it still cannot include all the potential patients in our dataset whose first episode was a depressive episode and may later switch to mania/hypomania. Thirdly, although our results implicate that alteration of putamen and SFG function may appear in early stage of BD and MDD respectively, we could not determine whether these functional abnormalities exist before or after illness onset. In future work, follow up of at-risk subjects (e.g., offspring or first-degree relatives of the patients with mood disorders) would be helpful to clarify this issue.

## Conclusions

In summary, we found altered fALFF of the putamen and SFG in depressive patients who were in their first episode, which may respectively correspond to the possibility of developing BD or remaining diagnosis of MDD at least 5 years after the onset of illness. The impairments of resting-state function in the putamen and SFG may serve as biomarkers for early identification of BD and MDD. These results implicate that abnormalities of key regions in the striatum and prefrontal areas may be trait markers for BD and MDD respectively.
